# The principle of the 3Rs between aspiration and reality

**DOI:** 10.3389/fphys.2022.914939

**Published:** 2022-08-12

**Authors:** Augusto Vitale, Laura Ricceri

**Affiliations:** Center for Behavioural Sciences and Mental Health—Istituto Superiore di Sanità, Rome, Italy

**Keywords:** 3Rs principle, animal experiments, animal legislation, animal models, animal ethics

## Abstract

The Principle of the 3Rs is widely recognised as the methodological and ethical backbone of contemporary animal research. Different authors also stress the reciprocal links among the 3Rs, and how these often complement and reinforce each other. We very much agree with this point, but in this contribution we would like to raise some problems related to the application of the “3Rs”. There is an obvious link among “Replacement, “Reduction” and “Refinement”, but it is worth to notice also that each “R” has its own conceptual characteristics, as well as its own level of applicability. For example, a realistic “methodological inertia” has to be expected more in the case of “Replacement” than in the case of “Refinement”. This also leads to a second order of issues, and here we will offer our experience as projects evaluators. The “3Rs” differ also in the possibility to verify how are applied by the proponents of research protocols involving the use of animal models. Sometimes it appears that the application of the Principle still resolves itself in the use of formulaic sentences, from which it is difficult to really understand the reality of the laboratory decisional and procedural processes. However, the demanding characteristics of the “3Rs” can vary greatly, and this is something that has to be considered. We propose that a network, or a virtual platform, of evaluators could help both researchers and evaluators for a more satisfactory understanding and pragmatic application of the Principle of the 3Rs.

## Introduction

The “Principle of the 3Rs” is considered nowadays by many researchers “the way” to carry out scientific procedures using animal models in research. However, it took some time, since the publication of “The Principles of Humane Experimental Techniques”, by William Russell and Rex Burch in 1959 (Russell and Burch, 1959), for the “3Rs” to gain a prominent role in animal research. The Principle, as a matter of fact, was pretty ignored or dismissed at the time of its publication. For instance, an anonymous reviewer in the journal Veterinary Records suggested to leave the book on the shelf, and consult it occasionally just to oppose anti-vivisectionist claims. (see Kirk, 2018). Is it true that the volume made for a difficult reading, but difficult it was the mission Russell and Burch embarked on. The idea was to bridge a gap between two distinct cultures, that is, science and humanism that many authors considered at those times irremediably in conflict with each other (Snow, 1963). Russell and Burch, instead, thought that there were common features that could unite the two worlds, and that it was possible to engage in a significant interdisciplinary framework. This framework would have been instrumental in changing the science of laboratory animals, and the aim would have been to limit, if not to avoid altogether, the experience of negative mental states in a laboratory animals. In other words: another way of using animals in research laboratories was needed, towards a elimination or significant decrease of animal suffering. An important aspect of the Russell and Burch’s idea, was the effort to shift the problem of animal management and care from the laboratory technicians to the researchers themselves. This has been a crucial strategic move: it inspired the idea that animal welfare was directly related to scientific quality and therefore was in the scientists’ interests to care for it. This is still a strong argument today. To apply the “3Rs” to our daily activity as animal researchers is not just to satisfy important new ethical awareness on our relationship with other animals, but also to meet the need for a scientifically better animal science (and this is particularly true when the behaviour of the experimental subjects determines the results we are looking for (see, for example, Poole, 1997).

It is doubtless, however, that the “Principle” presents a distinct ethical flavour, but when linking the Russell and Burch’s Principle to an ethical dimension (not something the two authors really looked for originally) a distinction has to be made. If the ethical questions is whether is morally acceptable or not to use animals in research laboratories for our own advantage as human beings, “the “Principle” cannot help us because it was not originally formulated within a “animal rights” perspective. Instead, the “3Rs” have to do with an “animal welfare” consideration of the research animals. It acts on animals that are still used in laboratory research (perfectly in line with the Directive 2010/63/EU on the protection of animals used in scientific procedures). In this sense, to affirm that the “Principle” has failed because animals are still used in research (see for example, Ibrahim, 2006; Blattner, 2019) misses the point, in our opinion, in two ways: 1) it misses to understand the “Principle” as a unitary concept (there is not JUST “Replacement”); 2) it suggests that the “Principle” aim is just to eliminate animals from research, and if this is an ethical demand that would concern “animal rights” reasonings, rather than “animal welfare”, as illustrated above. On the first point, Russel and Burch write: “Desirable as replacement is, it would be a mistake to put all our humanitarian eggs in this basket alone. The progress of replacement is gradual, not is it ever likely to absorb the whole of experimental biology” (Russell and Burch, 1959, pag. 105). This sentence could have been written today and is, in our opinion, a further evidence of the current relevance of the “Principle”.

**FIGURE 1:**
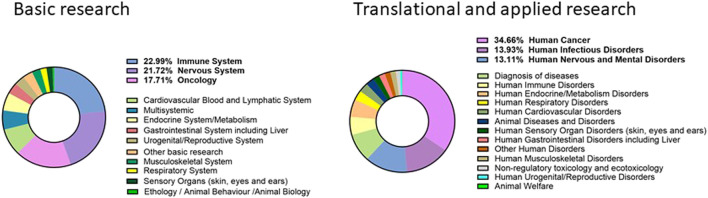
Use of laboratory mice in Europe (2019, Eu and Norway). Data from Alures database; in bold, percentages of the first three purposes for basic and translational research.

That replacement is gradual is also evident from data published on animals used for scientific purposes on the ALURES platform, published by the EU Commission.

(https://ec.europa.eu/environment/chemicals/lab_animals/alures_en.htm). As an example data concerning use of mouse species (the most common animal used in research and testing) in 2018 (the most recent database). While it could appear disappointing the relatively minor decrease in the number of animals used throughout the last 4–5 years, it is at the same time encouraging that the largest categories (half of the donut in Figure 1) for both basic and translational research focus on the most complex and integrated system, presumably those more difficult at the moment to model in *in vitro* systems.

## The application of the 3Rs principle

We have stressed the importance of considering the “Principle of the 3Rs” as a unitary tool to approach animal experiments in a more humane way, and how this is strictly related to the improvement of the quality of research itself.

**FIGURE 2:**
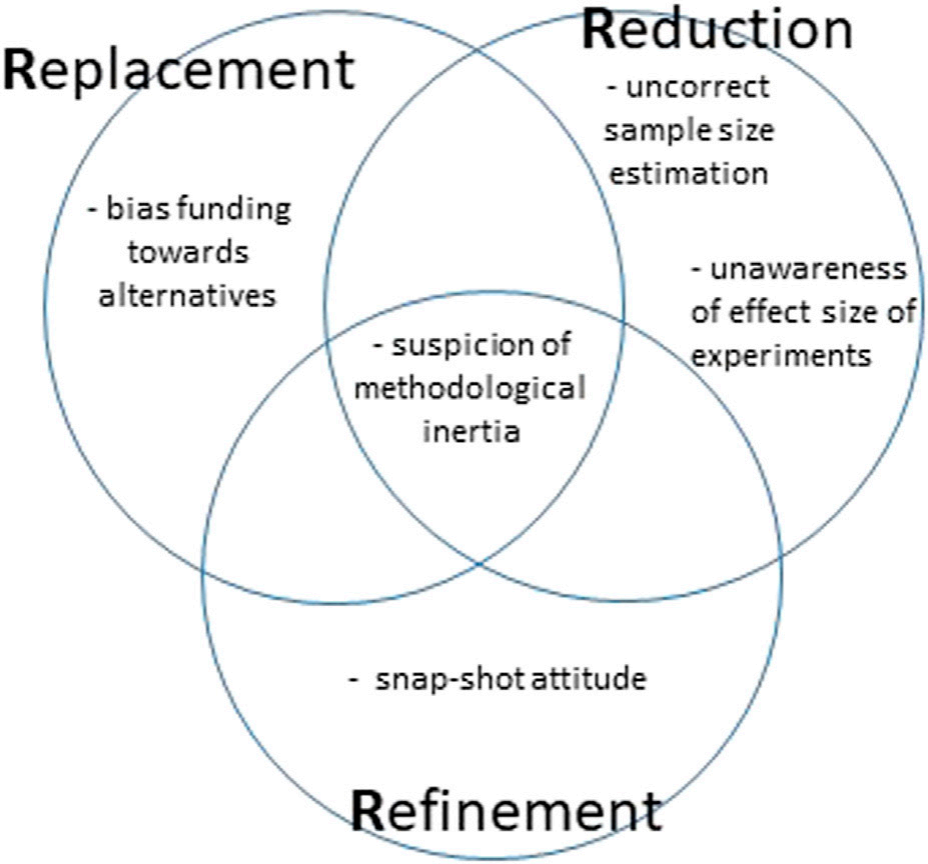
Potential issue troubling a correct application of the 3Rs Principle.

Having said that, are the “3Rs” equally applicable when devising a particular research protocol? In other words: are the different “Rs” requiring the same intellectual and practical efforts from the researchers? It seems, and this does not detract from the methodological validity of the Principle, that this is not the case. The “3Rs” have an order of appearance (Olsson et al.. 2011), and each one of them carries with it theoretical and practical problems as well as misuses (see next sections and Figure 2).

### Replacement

How much it takes to change model? One of the main obstacles in applying this particular concept is what we can define as “methodological inertia”.

Imagine a senior researcher, with a very good track record of publications and funding. This researcher has always used a particular animal model for his/her research, and his/her success is basically identifiable with that particular animal model. It is not so given for granted that this colleague would be ready to change all of his/her methodological routine (experimental techniques, animal housing and so on…) to replace such successful model. And on what basis? Is there a real added scientific value with the new model? Is it just an ethical decision? How long will it take to verify that the new model is actually working? All of these questions require time to be adequately addressed. The pressure imposed on researchers by the “to publish-to obtain funds-to publish” loop, the competitive way in which science mostly works today, does simply not leave enough time to re-think a successful research routine.

How can this situation be addressed? With care and understanding by the Animal Welfare Bodies, with courage by the researcher. The culture of change must be shared by the entire scientific community, composed by both proponents and evaluators of research projects (and often these two roles overlap).

In parallel, fundings should be devoted to those studies aimed at a frank comparison of *in vitro* and *in vivo* models, so still including use of animal models to evaluate which aspects of the model can currently be replaced by other non-animal system and which indeed cannot yet. The recent European Parliament resolution to accelerate the transition to innovation without the use of animals in research, regulatory testing and education (RSP, 2021) of September 2021 apparently does not support such a view. By contrast, it refers to mechanisms for the preferential funding of non-animal methods across all EU research and innovation initiatives, as such alternative methods bring additional costs and investment needs; points, therefore, to the need for increased and targeted funding under Horizon Europe for advanced non-animal models. Maybe a positive point is the reference made to medium- to long-term funding available to ensure the fast development, validation and introduction of alternative testing methods to replace animal testing methods, particularly for key toxicological endpoints, that is, indeed a wise and well-timed request. We believe that validation of new models is crucial and, primarily in the case of basic research, this means that funding should also be accessible to foster a deep interaction between researchers with different views and background, i.e., those dealing with animal models and those dealing with alternative systems. Unfortunately, the response by the European Commission to the European Parliament resolution (SP, 2021) does not address this possibility, but rather it barely defends the efforts by the Commission in funding research on alternative methods to the use of animals.

### Reduction

Application of Reductions refers to methods that minimise the number of animals used per experiment or study, achieving the same scientific objective. More recently, reduction approaches also include methods that maximize information gathered per animal (to reduce the use of additional subjects). It is probably simple for evaluators to identify Reduction strategies in a particular research protocol, provided that statistical and experimental details are provided; it remains essential (and sometime more difficult) for researchers to appropriately design their studies to ensure robust and reproducible findings. Whereas reproducibility issues may induce to inflate the sample size, Reduction principle should drive the researcher to choose the minimum values among those considered appropriate for robust results.

The reproducibility debate about *in vivo* research (Munafò et al., 2022) has somehow updated the role of Reduction as the necessary point of *equilibrium* between two opposite forces, namely ethical reduction in the number of animals used on one side, and increasing number of subjects to achieve statistical power on the other.

### Refinement

In our opinion there is always space for Refinement actions. However, if to apply Refinement measures means to reduce the level of sufferance of the animals used in a particular research protocol, one of the *condition sine qua non* is to be able to identify the potential and actual level of sufferance the experimental subjects are experiencing, Unfortunately, we believe that our perception of what sufferance is of a particular individual belonging to a particular species is still too sketchy. As an example of this difficulty, Borgi and colleagues have collected the opinion of researchers (from different countries and fields of application) on the acceptability (expressed in different degrees) of different species to be subjected to severe procedures. The answer was given based on the scientists’ knowledge on the level of sufferance a particular species could experience, and not on personal or ethical grounds. The results showed a significantly diversified perception of sufferance comparing different species, but also within the same species, for different researchers (Borgi et al., 2021). In other words: different researchers had different perception of what sufferance is for a mouse, or a rat, or an octopus. It appears that more research and thinking have to be dedicated to this aspect.

But how, in the meantime, can approach this difficulty? We think that we will have to continue for the time being to deal with a certain degree of uncertainties on how to classify animal sufferance. This uncertainty will continue to come from both objective difficulties related to interpret behavioural signs of sufferance, as well as the researchers’ personal differences in evaluating those signs. However, more “objective” ways are already available. For example, in our experience now proponents of project proposals must include a table, with a scoring system. The table is used to determine humane end-points, and it evaluates the physical and behavioural conditions of the individual animal. The total score, obtained summing up the score for different items (such as, condition of the fur, spontaneous behaviour…) will determine the humane end-point (the higher the score, the more suffering is the animal) (Table 1 illustrates an example modified from a project proposal in rodents).

**TABLE 1:** Standardized scores for the assessment of suffering and the determination of “humane endpoint” in rodents.

Parameter	Condition of the animal	Score	Date/Time
Appearance	Normal	0	
	Appearance Normal Poor hygiene (persistent grooming) indicating a slight depression of the sensory system	1	
	Shaggy coat	2	
	Shaggy coat and/or kyphosis; redness of eyes and nose	3	
	Persistent immobility	4	
Bodily functions	Normal	0	
	Decrease in body weight and/or food intake <5%	1	
	Decrease in body weight and/or food intake <15%	2	
	Decrease in body weight and/or food intake <20%	3	
Breath frequency	Normal frequency	0	
	Slight alterations	1	
	Increased rate and abdominal breathing	2	
	Decreased rate and abdominal breathing	3	
	Marked abdominal breathing and cyanosis	4	
Spontaneous behaviour	Normal	0	
	Slight alterations, excitability (in the case of a test of the auricle or of the pinch in the paw)	1	
	Isolated from the others, persistent inactivity	2	
	Restless or almost motionless; compulsive behaviors; repeated circular movements (stereotypies)	3	
Environment	Normal (nest built)	0	
	Nest just partially built	1	
	No nest present	2	
	Diarrhea	3	
Other	Ears turned outwards and/or backwards; sharp muzzle; narrow and half-closed eyes	4	
Total score			

Outcomes: 0–4, normal; 5–9, condition that requires daily monitoring; 10, animal with initial signs of distress for which indication by the designated veterinarian is required; 11–12: animal with signs of suffering for which indication by the designated veterinarian is required; 13, End-point.

Furthermore, in literature it is now possible to find “grimace scales”, that is, a series of pictures portraying mouse facial expressions, associated with different degrees of sufferance, a useful tool to better identify the intensity of pain and discomfort experienced by experimental mice (Langford et al., 2010).

## Discussion—In search of the 3Rs (non In search OT the 3Rs)

We believe that the culture of the “3Rs” can be enforced mainly in two ways, and we refer here to our experience as project evaluators within the Italian system. Quick and fast application of the Principle comes from our activity as reviewers of project applications. Questions and requests for modifications of the research application, in the spirit of the “3Rs” are proposed to the researchers resulting, hoping so, in better protocol (both in terms of science and animal welfare). This action is pretty practical from our points of view. However, it does not always automatically translates in more awareness by the researchers of what the Principe is about. On one hand, the risks is that the proponents just apply the required modification to have the submission approved; on the other us, evaluators, do not find the time to engage in a proper dialogue with the proponents because the number of project to review can be overwhelming. In this context, it would be very useful to realise a forum or an online platform, on which evaluators from different institution and countries would be able to seek advice and exchange experiences in the application of the Principle of the “3Rs” by research proponents.

The second way is to continue to spread the “3Rs” philosophy through courses, seminars and university lectures. This is an action aimed at slowly and progressively changing the culture of doing research with animals, primarily targeting younger researchers. But in doing so, we do not have to be shy about the limitations, both theoretically and practically speaking, of Russell and Burch’s idea. The recent Italian Leg. Decree issued in September 2021 (https://www.gazzettaufficiale.it/eli/id/2021/09/23/21A05569/sg) will help in this direction. It is now required by law that personnel included in a project proposal has knowledge and experience in the topics listed in the Annex V of the Leg. Decree 26/2014 (as required by the EU Directive 2010/63). Such skills (which have to be regularly up-dated) has to be documented by certification obtained by participating in specific courses.

It is not always possible to apply the Principle in all of its three aspects, but this does not absolutely mean that the Principle as an idea is neither obsolete nor ineffective. William Russell and Rex Burch spot it right!

## Data Availability

Publicly available datasets were analyzed in this study. This data can be found here: https://ec.europa.eu/environment/chemicals/lab_animals/alures_en.htm.
